# Cannabis and Psychedelics Among U.S. Young Adults: Use, Messaging Exposure, Perceptions, and Legalization Support

**DOI:** 10.3390/ijerph23020255

**Published:** 2026-02-17

**Authors:** Carla J. Berg, Cassidy R. LoParco, Darcey M. McCready, Laura C. Schubel, Patricia A. Cavazos-Rehg, Erin Kasson, Shriya Thakkar, Diane M. Ndisebuye, Y. Tony Yang

**Affiliations:** 1 Department of Prevention and Community Health, Milken Institute School of Public Health, George Washington University, Washington, DC 20037, USA; cassidy.loparco@gwu.edu (C.R.L.); darcey.mccready@gwu.edu (D.M.M.); lschubel@gwu.edu (L.C.S.); shriyat@gwu.edu (S.T.); mukundwadianen@gwu.edu (D.M.N.); 2 George Washington Cancer Center, George Washington University, Washington, DC 20052, USA; 3 Department of Psychiatry, Washington University School of Medicine, St. Louis, MO 63110, USA; pcavazos@wustl.edu (P.A.C.-R.); erinmkasson@wustl.edu (E.K.); 4 School of Nursing, George Washington University, Washington, DC 20006, USA; ytyang@gwu.edu

**Keywords:** cannabis, marijuana, psychedelics, hallucinogens, risk factors, mental health, perceptions

## Abstract

**Highlights:**

**Public health relevance—how does this work relate to a public health issue?**

- Cannabis and psychedelic use may have certain psychical and/or mental health benefits but also pose significant risks.
- The attitudes a population has about these substances plays a significant role in shaping public health policy and thus has implications for population health impact.

**Public health significance—why is this work of significance to public health?**

- Current findings underscore the importance of disseminating information about the potential benefits and risks, given the strong associations between perceptions and use of these products and their legalization among young adults.
- This is particularly crucial given that mental health was associated with substance use and legalization support, as information about their potential therapeutic use and benefits is limited.

**Public health implications—what are the key implications or messages for practitioners, policy makers and/or researchers in public health?**

- Perceptions of these products are important for understanding their use and legalization support, and information about psychedelics is especially important at this pivotal time.
- Policy and public health should pair trauma-informed prevention and harm-reduction with balanced, evidence-based communication as jurisdictions weigh psychedelic policy.

**Abstract:**

This study assessed the correlates of cannabis and psychedelic use and legalization support among young adults in the United States (US). Using 2025 data among adults ages 18–34 (*n* = 3227), we assessed cannabis and psychedelic message exposure and perceptions, mental health symptoms (Patient Health Questionnaire-4 [PHQ-4]), and adverse childhood experiences (ACEs) in relation to past-6-month cannabis use (40.5%), past-year psychedelic use (11.9%), and legalization support. Relative to cannabis, psychedelics showed less legalization support, promotional and risk-message exposure, and social acceptability and higher perceived addictiveness and harm (*p*’s < 0.001). Factors associated with cannabis use and greater legalization support included: lower perceived addictiveness (aOR = 0.88, CI = 0.83–0.93; B = −0.04, SE = 0.01) and harm (aOR = 0.75, CI = 0.71–0.80; B = −0.16, SE = 0.01), higher social acceptability (aOR = 1.25, CI = 1.19–1.33; B = 0.19, SE = 0.01), and higher PHQ-4 (aOR = 1.04, CI = 1.01–1.07); more ACEs (aOR = 1.10, CI = 1.06–1.14) and more promotional (aOR = 1.08, CI = 1.01–1.17) and risk-message exposure (aOR = 1.27, CI = 1.17–1.39) were associated with use. Factors associated with psychedelic use and greater legalization support included: more promotional-message exposure (aOR = 1.61, CI = 1.36–1.91; B = 0.09, SE = 0.04), lower addictiveness (aOR = 0.87, CI = 0.78–0.97; B = −0.03, SE = 0.02) and harm (aOR = 0.74, CI = 0.66–0.82; B = −0.19, SE = 0.02), higher acceptability (aOR = 1.59, CI = 1.47–1.73; B = 0.15, SE = 0.01), and higher PHQ-4 (aOR = 1.06, CI = 1.02–1.11; B = 0.02, SE = 0.01); more risk-message exposure (aOR = 1.29, CI = 1.08–1.54) and ACEs (aOR = 1.15, CI = 1.09–1.21) were associated with use. Perceptions and mental health may influence cannabis and psychedelic use and legalization support, and message exposure may be particularly relevant in shaping psychedelic use and legalization support. Thus, information is crucial to ensure population understanding of the risks, benefits, and overall population impacts of cannabis and psychedelic use and legalization.

## 1. Introduction

Psychedelics include classic serotonergic hallucinogens (e.g., lysergic acid diethylamide [LSD or “acid”], psilocybin [“magic mushrooms”]), and dissociative agents such as ketamine that can produce mood and cognitive alterations and hallucinations [1,2]. Every year, millions of people around the world use psychedelics [3,4,5]. In the United States (US), psychedelic use prevalence has steadily risen over the past decade [2,3,6], from 1.0% of US adults reporting past-year use in 2014 [7] to 3.6% in 2024, with particularly high rates among those ages 18–25 (6.8%), 26–29 (9.6%), and 30–34 (6.2%) [8].

Research on psychedelic benefits and risks has yielded mixed results [5,9,10,11,12,13,14,15]. Some studies indicate therapeutic benefits for treating various mental health conditions (e.g., major depressive disorder, substance use disorders) and/or longer-term impacts of experiencing a history of trauma (e.g., post-traumatic stress disorder [PTSD]), such as trauma related to adverse childhood events (ACEs) [5,9,10,11,12,13,14,16,17]. Furthermore, some consider self-administered psychedelics as more effective in managing symptoms than other medical treatments [5,18]. However, therapeutic promise exists alongside risks (e.g., misuse, addiction, other mental health conditions), especially when not clinically controlled [15,19,20,21,22,23,24,25,26,27,28]. It remains unclear whether these associations indicate that psychedelic use contributes to these outcomes—or if individuals use psychedelics to treat mental health and substance use disorders [27]. Thus, further research is necessary [29,30,31].

Notably, the 1971 United Nations’ Convention on Psychotropic Substances categorized psychedelics as Schedule 1 substances in 197 countries [32], making recreational psychedelic use nearly globally prohibited [32,33]. However, renewed interest in therapeutic use has led some countries (e.g., Australia, Israel, Canada) to permit psychedelics for medical purposes. Under US federal law (the Controlled Substances Act [CSA], passed in 1970), most psychedelics are classified as Schedule I substances (i.e., no medical use, high potential abuse), but some with mild psychedelic effects are classified differently (e.g., ketamine is Schedule III) [34,35,36]. However, state laws on psychedelics vary [37]. Two states have decriminalized and created regulatory frameworks for psychedelics: (1) in 2020, Oregon legalized psilocybin for therapeutic purposes (e.g., mental health conditions); and (2) in 2022, Colorado legislation created a framework for psychedelic regulation and legalization (covering psilocybin and psilocin offered in “healing centers”) [37]. Certain states have reduced penalties for growing or using psychedelics, some allow medical research, and others are developing/considering new legislation [37]. Within this context, use prevalence has shown particular increases in decriminalized states (i.e., Oregon, Colorado: 3.3% in 2019–2020 to 5.4% in 2021–2023) vs. non-decriminalized states (2.4% to 2.8%) [38].

Psychedelics’ shifting regulatory context is reminiscent of shifts for cannabis in the US. Like psychedelics, cannabis has some medical uses (e.g., pain, muscle spasticity, nausea/vomiting, seizures), but also risks (e.g., use disorder, psychosis, mood disorders, cognitive impairment) [39]. Furthermore, the 1970 CSA also categorized cannabis as a Schedule 1 substance. Recognizing potential medical benefits, in 1996, California became the first state to legalize medical use. By December 2025, 40 states, DC, Puerto Rico, Guam, and the US Virgin Islands, have laws allowing medical use [40]. States have also legalized nonmedical (i.e., ‘recreational’) cannabis, starting in 2014 in Colorado and Washington; as of 2025, 25 states fully legalized cannabis for both nonmedical and medical use [40]. Notably, use prevalence has increased over the past decade, from 12.1% of US adults reporting past-year cannabis use in 2014 [7] to 23.4% in 2024, with particularly high rates among those ages 18–25 (35.0%), 26–29 (37.7%), and 30–34 (31.7%) [8].

Perceptions of these products are important for understanding their use and the extent to which the population will support continued legal expansion. One UK-based study found that viewing drug use as a health issue, rather than a criminal issue, predicted lower perceived risk and greater psychedelic legalization support [41]. US-based studies found that psychedelic use was perceived as posing a range of psychospiritual benefits (e.g., enhanced life meaning, spirituality) but more risks (e.g., bad “trips,” accidents, impaired judgment, legal problems) [42], and that recreational use was perceived as having negative health consequences while therapeutic use was perceived as having positive health consequences [43]. Similarly, cannabis perceptions (e.g., risks, social acceptability) and interest in its medical utility in the US have generally become more favorable over the past decade [44,45,46,47,48], which have coincided with increased support for cannabis legalization [44,49].

Perceptions and attitudes are shaped by exposure to information. A US-based survey found that prominent sources of adults’ information about psychedelics were their own experiences (80%), online (e.g., websites 62%, discussion forums 57%), friends (61%), books (57%), and scientific articles (55%) [50]. Similarly, studies have documented various cannabis information sources communicating both benefits (e.g., advertising, retailers, friends/family, online) and risks (e.g., public health campaigns, warning labels, friends/family, online) [51,52,53,54,55]. Unfortunately, many of these sources may include misinformation—either intentionally or unintentionally [56,57].

Given the parallels between psychedelics and cannabis and the more recent shifts in the psychedelic regulatory and use context, it is crucial to examine attitudes and use outcomes that can inform legislative, regulatory, and prevention efforts. Factors associated with use and support for decriminalization/legalization may be similar across these substances and thus are important to understand [58]. One relevant theoretical framework for understanding how populations form opinions about ideas over time is diffusion of innovation (DOI) theory [59,60,61]. DOI posits that communication is key to shaping opinions and that support increases as the proportion of the population that favorably perceives the idea increases and expands communication about potential benefits [59,60,61]. This theory also suggests the importance of exposure to relevant information (both favorable and unfavorable), as these exposures influence perceptions and attitudes, and ultimately behavior [59,60,61]. Thus, factors associated with use and support for decriminalization/legalization likely include exposure to information, highlighting both benefits and risks, as well as perceived risk and social acceptability. Additionally, given these substances’ potential therapeutic utility, whether individuals experience mental health symptoms or historic trauma like ACEs may also impact their perceptions of these substances [5,9,10,11,12,13,14,16,17,39]. This study assessed cannabis and psychedelic use, use intentions, and legalization support, as well as potential correlates (mental health, ACEs, promotional and risk-message exposure, perceived risk and social acceptability). Message exposure, perceptions, use intentions, and legalization support for cannabis vs. psychedelics were also compared.

## 2. Materials and Methods

### 2.1. Study Overview

This study used survey data (collected in February–May 2025) among young adults participating in the Cannabis Regulation, Marketing, and Appeal (CARMA) study. CARMA is a longitudinal investigation of nonmedical cannabis retail, marketing, and use that launched in 2023 and involves 5 waves of survey data, each 6 months apart [62]. The study was approved by the George Washington University Institutional Review Board.

### 2.2. Participants and Recruitment

From June–November 2023, advertisements targeting eligible young adults (English-speaking US residents ages 18–34, given that this age group shows the highest cannabis use [and also psychedelic use] [8]) were posted on Facebook. After clicking on advertisements, individuals were messaged via chatbot on Facebook Messenger (which verified each individual had a Facebook account and precluded duplicate chatbot interactions). The chatbot provided an abbreviated study overview, assessed key factors (age, state of residence, race, ethnicity, sex, past-month cannabis use), and provided individuals deemed preliminarily eligible a unique link to the study webpage which expired after a single use. There, formal consent was obtained, eligibility was confirmed, and the baseline survey was administered. Participants were told that the study required a valid email address and phone number and confirming their participation by clicking a link in an email sent 7 days post-baseline survey (which allowed study staff to verify contact information and review survey data for logical responses, etc.). After confirming, they received their incentive ($10 Amazon e-gift card). Purposive, quota-based recruitment was used to ensure representation of key subgroups (i.e., ~50% past-month cannabis use, ~50% males and females, ~40% racial/ethnic minorities) to power subgroup analyses.

Overall, 6908 individuals completed chatbot pre-screening, 6128 (88.7%) were preliminarily eligible, 5827 (95.6%) visited the study webpage, 5672 (97.3%) were consented and eligible, 4385 (77.3%) completed the survey and were sent confirmation emails, and 4031 (91.9%) confirmed their participation and were enrolled in the study. Current analyses focused on the 3240 (80.4%, *n* = 3240/4031) participants who completed the Wave 4 (W4) survey (February–May 2025), which included unique items assessed only at W4 (e.g., psychedelic-related questions). We further focused our sample on those participants who had complete data on measures included in these analyses (*n* = 3227/3240, 99.6% of W4 participants).

### 2.3. Measures

All measures were collected at W4, except for sociodemographic characteristics, which were assessed at W1.

#### 2.3.1. Dependent Variables

At each wave, cannabis use was assessed by asking, “This question refers to marijuana, also known as cannabis, pot, weed, etc., including dried herb, edibles, oils, hash/kief, concentrates, beverages, tinctures, lotions, etc. (Do not include hemp-derived cannabinoids, like Delta-8-THC, etc.) In the past 6 months, how many days did you use cannabis?” Past-6-month use was assessed at each wave to account for time between assessments [63,64].

At W4, we assessed psychedelic use by asking, “Psychedelics are drugs that can temporarily alter a person’s mood, thoughts, and perceptions. Have you ever used: ‘acid’ (LSD); ‘magic mushrooms’ (Psilocybin, Amanita); ‘ecstasy/molly’ (MDMA); ketamine; DMT; tianeptine (aka Neptune’s fix); salvia; or another psychedelic” [63]. Participants who reported ever using magic mushrooms were asked the type of mushrooms (Psilocybin, Amanita) [63]. For each substance participants reported ever using, participants were asked, “In the past 12 months, on how many days did you use [product]?” [63]. Past-year use was assessed to compare use rates in our sample with those in other studies (e.g., National Survey on Drug Use and Health) [8]. A variable was created to indicate any vs. no past-year psychedelic use.

Cannabis and psychedelic use intentions were assessed by asking, “How likely are you to try or continue to use each of the following products in the next year?” with regard to: marijuana; ‘acid’ (LSD); ‘magic mushrooms’ (Psilocybin/Amanita); ‘ecstasy/molly’ (MDMA); and ketamine (1 = not at all to 7 = extremely) [63]. An index score summarizing psychedelic use intentions was created by taking the average of responses for the 4 psychedelics (Cronbach’s α = 0.82).

Cannabis and psychedelic legalization support was assessed by asking participants to rate their level of agreement with: (1) “The use of [marijuana; psychedelics] for justified medical reasons should be legal at the federal level”; (2) “The use of [marijuana; psychedelics] for recreational purposes should be legal at the federal level” (1 = strongly disagree to 5 = strongly agree; average across 2 items calculated as summary score for each substance; Cronbach’s α = 0.82 for each) [44,65,66].

#### 2.3.2. Independent Variables

Mental health symptoms were assessed using the Patient Health Questionnaire-4 (PHQ-4) [67], which includes 2 items assessing past 2-week depressive symptoms and 2 for anxiety (0 = not at all to 3 = nearly every day). This variable was used as a continuous sum score (range: 0–12; Cronbach’s α = 0.89). ACEs were assessed at W1 using the ACEs—10 item scale, which assesses maltreatment and household challenges before the age of 18 (0 = no, 1 = yes); this variable was also treated as a continuous sum score (range: 0–10; Cronbach’s α = 0.81) [68].

Promotional-message exposure was assessed by asking, “In the past 6 months, how often have you noticed advertisements or promotions (online; in stores/kiosks; outdoor signs, billboards, TV/movies, radio; newspapers/magazines; mail/email/text) for: (1) marijuana; or (2) psychedelics” (0 = not at all; 1 = less than once/month; 2 = 1–3 times/month; 3 = 1–3 times/week; 4 = daily or almost daily; 5 = more than once/day) [51,69]. If participants reported any exposure to such messages for psychedelics, they were asked which psychedelic was featured. Risk-message exposure was assessed by asking, “In the past 6 months, how often have you heard or seen information, for example, in educational or public health campaigns, or news stories about risks related to…” with regard to the same products (using the same response options), with the same follow-up question to indicate psychedelic(s) addressed [51,69].

Participants were also asked about product perceptions, specifically, “How [addictive; harmful to your health; socially acceptable] do you think the use of the following products are? marijuana; ‘acid’ (LSD); ‘magic mushrooms’ (Psilocybin, Amanita); ‘ecstasy/molly’ (MDMA); and ketamine” (1 = not at all to 7 = extremely) [69]. An index score summarizing each perception for psychedelics was created by taking the average of responses for the 4 psychedelics (Cronbach’s α = 0.85, 0.85, and 0.87).

#### 2.3.3. Covariates

Sociodemographics included age, sex at birth, sexual orientation, ethnicity, race, education level, relationship status, parental status, and community type (rural, micropolitan/suburban, metropolitan/urban). Participants’ reports of state of residence were coded for state cannabis regulatory context (no legal cannabis, medical legal, nonmedical/medical legal).

### 2.4. Data Analysis

Descriptive analyses characterized participants’ sociodemographics, cannabis and psychedelic use status and intentions, legalization support, mental health, ACEs, promotional and risk-message exposure, and perceptions overall and by past-6-month cannabis use and past-year psychedelic use status. We also assessed correlations among mental health, ACEs, message exposure, perceptions, use intentions, and legalization support for cannabis and psychedelics (see Supplementary Table S1).

Two multivariable binary logistic regression models assessed sociodemographics, mental health, ACEs, message exposure, and perceptions in relation to past-6-month cannabis use and past-year psychedelic use, including cannabis-specific message exposure and perceptions for cannabis and psychedelic-specific message exposure and perception for psychedelics. Then, 4 multivariable linear regression models assessed these factors in relation to next-year cannabis and psychedelic use intentions and legalization support (controlling for use status). All analyses were conducted using SPSS v27 (IBM, Armonk, NY, USA), and significance was set at *p* < 0.05.

## 3. Results

### 3.1. Participant Characteristics

As shown in Table 1, participants were age 26.38 (SD = 4.77) on average, 38.7% male, 27.7% sexual minority, 19.1% Hispanic, 65.5% White, 12.5% Black, 15.1% Asian, 6.8% other race, 52.6% < bachelor’s degree educated, 41.2% married/cohabitating, 29.8% parents, and 49.7% metropolitan/urban.

### 3.2. Comparisons of Cannabis vs. Psychedelic Use and Related Factors

Overall, 40.5% (*n* = 1307) reported past-6-month cannabis use. Past-year use of any psychedelic was reported by 11.9% (*n* = 385), with the most endorsed being psilocybin/amanita (8.6%), MDMA (4.3%), and LSD (3.5%). Notably, 69 participants (2.1% of the W4 sample) reported past-year psychedelic use but no past-6-month cannabis use, 991 (30.7%) reported past-6-month cannabis use but no past-year psychedelic use, and 316 (9.8%) used both (Table 1).

Use intentions were higher for cannabis vs. all psychedelics (M = 3.34, SD = 2.51 vs. M = 1.55, SD = 1.21, *p* < 0.001; Table 1). Supplementary Table S2 displays next-year use intentions for cannabis and each psychedelic assessed, as well as for the overall psychedelic index score, showing that 50.1% of the sample reported higher use intentions for cannabis (43.9% equal, 5.5% higher for psychedelics). Of the psychedelics, use intentions were highest for psilocybin/amanita (M = 1.76, SD = 1.57 vs. M = 1.55, SD = 1.21 across all psychedelics).

Legalization support was higher for cannabis than psychedelics (*p*’s < 0.001; Table 2). Furthermore, compared to cannabis, only 8.0% were more supportive of legalizing psychedelics (26.0% equal, 66.0% less; Supplementary Table S2).

Promotional- and risk-message exposure was higher for cannabis vs. psychedelics (*p*’s < 0.001), and on average, psychedelics (vs. cannabis) were perceived as more addictive, more harmful, and less socially acceptable (*p*’s < 0.001; Table 2). Supplementary Table S2 shows perceptions for cannabis and each psychedelic assessed, as well as for the overall psychedelic index score. Relative to cannabis, 45.7% perceived psychedelics as more addictive (19.7% equal, 34.6% less), 88.1% more harmful (15.4% equal, 11.9% less), and 82.4% less socially acceptable (13.2% equal, 4.3% more; Supplementary Table S2). Notably, across psychedelics and cannabis, psilocybin/amanita was perceived as least addictive (M = 4.15, SD = 2.03 vs. M = 4.54, SD = 1.78 across all psychedelics and M = 4.24, SD = 1.85 for cannabis), and among the psychedelics, psilocybin/amanita was perceived as least harmful (M = 4.59, SD = 1.98 vs. M = 5.12, SD = 1.60 for all psychedelics) and most socially acceptable (M = 3.13, SD = 1.95 vs. M = 2.73, SD = 1.66 for all psychedelics, *p*’s < 0.001; Supplementary Table S2).

### 3.3. Bivariate Analyses Assessing Factors Associated with Cannabis and Psychedelic Use

Table 1 shows bivariate results assessing sociodemographics associated with past-6-month cannabis use (older age, sexual minority, Hispanic, White vs. Asian, <bachelor’s degree educated, having children) and past-year psychedelic use (older, male, sexual minority, White vs. Asian, urban). Table 2 shows bivariate results assessing support, psychosocial factors, message exposure, and perceptions in relation to past-6-month cannabis use status and past-year psychedelic use status. Factors related to both use outcomes included greater support and risk minimization, more mental health symptoms and ACEs, greater promotional and risk-message exposure, lower perceived addictiveness and harm, and greater perceived social acceptability pertaining to both cannabis and psychedelics.

### 3.4. Multivariable Analyses Assessing Correlates of Cannabis Use, Intentions and Legalization Support

As shown in Table 3, factors associated with past-6-month cannabis use included being in a state with legal nonmedical cannabis (vs. no legalized cannabis), older, male, sexual minority, Black (vs. White), White (vs. Asian), <bachelor’s degree educated, and urban (vs. rural), as well as reporting more mental health symptoms and ACEs, more promotional- and risk-message exposure, lower perceived addictiveness and harm, and higher perceived social acceptability. As shown in Table 4, correlates of cannabis use intentions included identifying as sexual minority and White (vs. Black) and reporting lower perceived addictiveness and harm, higher perceived social acceptability, and past-6-month use. Factors associated with cannabis legalization support included being a sexual minority, White (vs. Asian), ≥bachelor’s degree educated, and suburban (vs. rural), and reporting more mental health symptoms, lower perceived addictiveness and harm, higher perceived social acceptability, and past-6-month use (Table 4).

### 3.5. Multivariable Analyses Assessing Correlates of Psychedelic Use, Intentions and Legalization Support

As shown in Table 3, correlates of past-year psychedelic use included being male, Black (vs. White), and urban (vs. rural), as well as reporting more mental health symptoms and ACEs, more promotional- and risk-message exposure, lower perceived addictiveness and harm, and higher perceived social acceptability. As shown in Table 5, factors associated with psychedelic use intentions included being male, Black (vs. White), <bachelor’s degree educated, and parents, as well as reporting more ACEs, more promotional- and risk-message exposure, lower perceived harm, higher perceived social acceptability, and past-year use. Correlates of psychedelic legalization support included being older, male, and sexual minority, as well as reporting more mental health symptoms, more promotional-message exposure, lower perceived addictiveness and harm, higher perceived social acceptability, and past-year psychedelic use (Table 5).

## 4. Discussion

In this sample of US young adults, representing ~40% past-6-month cannabis use, 11.9% reported past-year psychedelic use (most commonly psilocybin/amanita). These rates are higher than national past-year use rates in this age group (cannabis: ~34.8%; psychedelics: ~7.5%) [8], likely due to purposive sampling of ~50% reporting baseline cannabis use. Psychedelic perceptions were less favorable than cannabis perceptions, likely related to the longer-standing cannabis legality in certain states [70], as DOI may suggest—given that exposure to relevant information (both favorable and unfavorable) over time is necessary to shape perceptions and behaviors [59,60,61].

Interestingly, while perceptions were associated with all use and legalization support outcomes, message exposure was only associated with one cannabis-related outcome—use, likely because those who use cannabis may be exposed to more advertising and information at points-of-sale, as well as warnings on advertisements and products [64]. Meanwhile, promotional-message exposure was associated with psychedelic use, use intentions, and legalization support, which may reflect that these information sources are more important early in the diffusion process than later, as people have more established perceptions based on their individual experiences [59,60,61]. Additionally, risk-message exposure was associated with psychedelic use, likely for the same reasons that risk-message exposure was associated with cannabis use [64].

Reporting more mental health symptoms was associated with cannabis use and legalization support and with psychedelic use and legalization support. Additionally, reporting more ACEs was associated with cannabis use and with psychedelic use and use intentions. This may reflect some studies that point to potential therapeutic benefits for treating certain mental health conditions, including depression, anxiety, and PTSD—both for psychedelics [5,9,10,11,12,13,14,16,17] and cannabis [39], and that some may view drug use as a health (vs. criminal) issue, which may increase legalization support [41].

Notably, state cannabis laws were only associated with one outcome—cannabis use, which may suggest that other factors play a more powerful role. Various sociodemographic factors showed anticipated associations, based on prior research documenting use rates or support among certain subgroups [8,41,71]. For example, factors associated with certain outcomes included being male (cannabis use, psychedelic use, intentions and legalization support), a sexual minority (cannabis use, intentions, and legalization support; psychedelic legalization support), Black (vs. White; cannabis use but lower intentions, psychedelic use and intentions), and White (vs. Asian; cannabis use and legalization support), <bachelor’s degree educated (cannabis use, psychedelic intentions), ≥bachelor’s degree educated (cannabis legalization support), and urban or suburban (vs. rural; cannabis and psychedelic use, cannabis legalization support).

### Limitations

This study is limited in generalizability, given social media-based recruitment and purposive sampling of ~50% young adults reporting past-month cannabis use. However, the goal of this study was to assess associations among variables rather than to estimate population prevalence or describe the general population’s sentiment about cannabis and psychedelics. Findings are also interpreted cautiously to attenuate related concerns. Additionally, self-reported measures introduce potential bias and are not inclusive of all potential mechanisms of cannabis and psychedelic related outcomes. Also, as noted in Section 2.3, past-6-month cannabis use was assessed at each wave of the study to cover the time period between assessments, while psychedelic use was only assessed at Wave 4 and used a past-year time frame to allow comparisons with other studies including national studies that use past-year timeframes. However, we acknowledge that the different time frames used for cannabis and psychedelic use (past-6-month vs. past-year) may have impacted findings (e.g., broader or more restricted timeframes may impact correlates of use). Finally, data were cross-sectional, precluding causal inference and preventing examination of temporal relationships between psychosocial factors and use patterns. Thus, future research using longitudinal designs and assessing a larger range of variables with representative samples is needed. Despite the limitations of this study, study strengths include the large sample size of young adults from all 50 states, which included a purposive sample proportion using cannabis, which allowed us to obtain perceptions across young adults who use cannabis and psychedelics and those who did not use. This study also included detailed assessments of various relevant factors, including message exposure, perceptions, mental health, ACEs, and key sociodemographics.

## 5. Conclusions

Perceptions and mental health may influence use and legalization support for cannabis and psychedelics among US young adults. Psychedelics were viewed as riskier and less acceptable, with less legalization support than cannabis. Furthermore, messaging exposure may be particularly key in shaping psychedelic use and legalization support during the early process of societal opinion formation. Thus, valid, evidence-based information on the potential risks, benefits, and population impacts of cannabis and psychedelic use and legalization is crucial to provide society the resources necessary to make informed decisions. This is particularly timely as jurisdictions weigh decisions to expand cannabis legalization and consider new psychedelic policy.

## Figures and Tables

**Table 1:** Characteristics of US young adults overall and by past-6-month cannabis use status and past-year psychedelic use status (*n* = 3227).

		Past-6-Month Cannabis Use			Past-Year Psychedelic Use		
	All *n* = 3227	No *n* = 1920 (59.5%)	Yes *n* = 1307 (40.5%)		No *n* = 2842 (88.1%)	Yes *n* = 385 (11.9%)	
Variable	M (SD) or *n* (%)	M (SD) or *n* (%)	M (SD) or *n* (%)	*p*	M (SD) or *n* (%)	M (SD) or *n* (%)	*p*
***Cannabis law*** (*n*, %)				0.209			0.224
No legal cannabis	652 (20.5)	408 (21.2)	246 (18.8)		584 (20.8)	68 (18.0)	
Medical cannabis legal	900 (27.9)	533 (27.7)	367 (28.0)		797 (28.0)	103 (26.8)	
Nonmedical cannabis legal	1675 (51.9)	981 (51.0)	699 (53.3)		1461 (51.4)	214 (55.6)	
***Sociodemographic factors***							
Age (M, SD)	26.38 (4.77)	26.17 (4.85)	26.71 (4.64)	0.002	26.30 (4.80)	26.96 (4.55)	0.011
Male (*n*, %)	1240 (38.7)	747 (39.1)	496 (38.1)	0.566	1045 (37.0)	195 (51.0)	<0.001
Sexual minority (*n*, %)	872 (27.7)	412 (22.1)	461 (35.6)	<0.001	742 (26.7)	130 (34.3)	0.002
Hispanic (*n*, %)	606 (19.1)	337 (17.8)	272 (21.0)	0.015	527 (18.8)	79 (20.8)	0.208
Race (*n*, %)				<0.001			<0.001
White	2041 (65.5)	1193 (64.3)	851 (67.2)		1811 (66.0)	230 (62.3)	
Black	390 (12.5)	184 (9.9)	206 (16.3)		323 (11.8)	67 (18.2)	
Asian	471 (15.1)	368 (19.8)	105 (8.3)		436 (15.9)	35 (9.5)	
Other	213 (6.8)	109 (5.9)	105 (8.3)		176 (6.4)	37 (10.0)	
Education < Bachelor’s (*n*, %)	1686 (52.6)	871 (45.7)	817 (62.5)	<0.001	1470 (52.1)	216 (56.1)	0.077
Relationship status (*n*, %)				0.381			0.451
Single/other	1899 (58.8)	1143 (59.5)	760 (57.9)		1669 (58.7)	230 (59.7)	
Married/cohabitating	1328 (41.2)	779 (40.5)	552 (42.1)		1173 (41.3)	155 (40.3)	
Parent/has children (*n*, %)	962 (29.8)	522 (27.2)	443 (33.8)	<0.001	853 (30.0)	109 (28.3)	0.267
Community type (*n*, %)				0.579			0.002
Rural	639 (19.8)	382 (19.9)	258 (19.7)		582 (20.5)	57 (14.8)	
Micropolitan/suburban	983 (30.5)	599 (31.2)	388 (29.6)		878 (30.9)	105 (27.3)	
Metropolitan/urban	1603 (49.7)	941 (49.0)	664 (50.7)		1380 (48.6)	223 (57.9)	
***Use status*** (*n*, %)							
Past-6-month cannabis use	1307 (40.5)	--	--	--	991 (34.9)	316 (82.1)	<0.001
Past-year psychedelics use	385 (11.9)	69 (3.6)	316 (24.2)	<0.001	--	--	--
***Use intentions*** (M, SD)							
Cannabis	3.34 (2.51)	1.79 (1.52)	5.60 (1.88)	<0.001	3.06 (2.44)	5.37 (2.04)	<0.001
Psychedelics ^	1.55 (1.21)	1.30 (0.97)	1.91 (1.42)	<0.001	1.34 (0.97)	3.06 (1.67)	<0.001

Notes: ^ Cronbach’s α for next-year use intentions (for LSD, psilocybin/amanita, MDMA, and ketamine) = 0.82. Lifetime use of any psychedelic was reported by 27.7% of participants, with the most commonly endorsed psychedelics being psilocybin/amanita (20.6%), MDMA (16.0%), and LSD (13.5%).

**Table 2:** Legalization support, psychosocial factors, message exposure, and perceptions overall and by past-6-month cannabis use status and past-year psychedelic use status (*n* = 3227).

		Past-6-Month Cannabis Use			Past-Year Psychedelic Use		
	All *n* = 3227	No *n* = 1920 (59.5%)	Yes *n* = 1307 (40.5%)		No *n* = 2842 (88.1%)	Yes *n* = 385 (11.9%)	
Variable	M (SD) or *n* (%)	M (SD) or *n* (%)	M (SD) or *n* (%)	*p*	M (SD) or *n* (%)	M (SD) or *n* (%)	*p*
***Legalization support*** (M, SD) ^a^							
Cannabis	3.88 (1.18)	3.54 (1.23)	4.39 (0.91)	<0.001	3.83 (1.20)	4.31 (0.95)	<0.001
Psychedelics	2.83 (1.24)	2.63 (1.19)	3.13 (1.24)	<0.001	2.72 (1.21)	3.67 (1.16)	<0.001
***Psychosocial factors*** (M, SD)							
PHQ-4—Mental health symptoms	3.61 (3.38)	3.39 (3.28)	3.95 (3.50)	<0.001	3.46 (3.32)	4.73 (3.64)	<0.001
PHQ-4—depressive symptoms	1.63 (1.73)	1.51 (1.67)	1.82 (1.82)	<0.001	1.55 (1.70)	2.20 (1.89)	<0.001
PHQ-4—anxiety symptoms	1.98 (1.90)	1.88 (1.85)	2.14 (1.97)	<0.001	1.91 (1.87)	2.54 (2.02)	<0.001
ACEs	2.70 (2.68)	2.20 (2.45)	3.45 (2.82)	<0.001	2.56 (2.61)	3.74 (2.93)	<0.001
***Past-6-month message exposure*** (M, SD)							
*Promotional-message exposure*							
Cannabis	1.32 (1.39)	1.16 (1.32)	1.55 (1.44)	<0.001	1.25 (1.36)	1.86 (1.44)	<0.001
Psychedelics	0.27 (0.74)	0.17 (0.57)	0.40 (0.92)	<0.001	0.18 (0.60)	0.86 (1.25)	<0.001
*Risk-message exposure*							
Cannabis	0.88 (1.17)	0.72 (1.05)	1.10 (1.29)	<0.001	0.81 (1.13)	1.34 (1.36)	<0.001
Psychedelics	0.26 (0.75)	0.17 (0.58)	0.40 (0.93)	<0.001	0.20 (0.64)	0.72 (1.22)	<0.001
***Perceptions*** (M, SD) ^b^							
*Addictiveness*							
Cannabis	4.24 (1.85)	4.54 (1.82)	3.80 (1.80)	<0.001	4.29 (1.85)	3.88 (1.78)	<0.001
Psychedelics	4.54 (1.78)	4.79 (1.77)	4.18 (1.74)	<0.001	4.64 (1.78)	3.79 (1.57)	<0.001
*Harm*							
Cannabis	3.55 (1.85)	4.05 (1.83)	2.82 (1.61)	<0.001	3.62 (1.86)	3.03 (1.68)	<0.001
Psychedelics	5.12 (1.60)	5.38 (1.56)	4.73 (1.58)	<0.001	5.26 (1.56)	4.07 (1.50)	<0.001
*Social acceptability*							
Cannabis	5.22 (1.93)	4.79 (2.06)	5.86 (1.49)	<0.001	5.14 (1.96)	5.80 (1.56)	<0.001
Psychedelics	2.73 (1.67)	2.44 (1.60)	3.16 (1.67)	<0.001	2.55 (1.59)	4.08 (1.57)	<0.001

Notes: Cronbach’s α for cannabis and psychedelics: ^a^ legalization support = 0.82 for cannabis and 0.82 for psychedelics. ^b^ Cronbach’s α for three psychedelic index scores (perceived addictiveness, harm, social acceptability) for LSD, psilocybin/amanita, MDMA, and ketamine = 0.85, 0.85, 0.87.

**Table 3:** Multivariable binary logistic regression models assessing sociodemographic, psychosocial factors, message exposure, and perceptions in relation to past-6-month cannabis use and past-year psychedelic use among US young adults (*n* = 3227).

	Past-6-Month Cannabis Use			Past-Year Psychedelic Use		
Variables	aOR	95% CI	*p*	aOR	95% CI	*p*
***Cannabis law*** (ref: no legal cannabis)						
Medical cannabis legal	1.29	1.00, 1.67	0.050	1.20	0.79, 1.81	0.395
Nonmedical cannabis legal	1.51	1.19, 1.91	0.001	1.42	0.98, 2.05	0.068
***Sociodemographic factors***						
Age	1.03	1.01, 1.05	0.004	1.02	0.99, 1.06	0.179
Male (ref: female)	1.28	1.06, 1.56	0.012	1.77	1.32, 2.38	<0.001
Sexual minority (ref: heterosexual)	1.30	1.06, 1.59	0.012	0.87	0.64, 1.18	0.375
Hispanic (ref: non-Hispanic)	1.14	0.89, 1.45	0.291	0.98	0.68, 1.42	0.919
Race (ref: White)						
Black	1.78	1.35, 2.34	<0.001	1.59	1.06, 2.39	0.025
Asian	0.73	0.54, 0.99	0.044	0.85	0.53, 1.36	0.504
Other	1.20	0.90, 1.61	0.220	1.35	0.88, 2.07	0.165
Education < Bachelor’s (ref: ≥Bachelor’s)	1.58	1.30, 1.92	<0.001	0.99	0.73, 1.33	0.933
Married/cohabitating (ref: single/other)	0.86	0.70, 1.06	0.154	0.94	0.70, 1.28	0.705
Parent/has child (ref: no)	1.27	1.00, 1.61	0.050	1.00	0.70, 1.44	0.983
Community type (ref: rural)						
Micropolitan/suburban	1.20	0.93, 1.54	0.169	1.30	0.84, 1.99	0.239
Metropolitan/urban	1.30	1.02, 1.67	0.036	1.52	1.02, 2.28	0.041
***Psychosocial factors***						
PHQ-4	1.04	1.01, 1.07	0.008	1.06	1.02, 1.11	0.007
ACEs	1.10	1.06, 1.14	0.000	1.15	1.09, 1.21	<0.001
***Past-6-month message exposure*** ^a^						
Promotional-message exposure	1.08	1.01, 1.17	0.032	1.61	1.36, 1.91	<0.001
Risk-message exposure	1.27	1.17, 1.39	<0.001	1.29	1.08, 1.54	0.005
***Perceptions*** ^a^						
Addictiveness	0.88	0.83, 0.93	<0.001	0.87	0.78, 0.97	0.012
Harm	0.75	0.71, 0.80	<0.001	0.74	0.66, 0.82	<0.001
Social acceptability	1.26	1.19, 1.33	<0.001	1.59	1.47, 1.73	<0.001
*Nagelkerke R-square*		*0.301*			*0.353*	

Notes: ^a^ For cannabis or psychedelics, respectively. aOR: adjusted odds ratio. CI: confidence interval.

**Table 4:** Multivariable linear regression models assessing sociodemographics, psychosocial factors, message exposure, and perceptions in relation to cannabis use intentions and legalization support among US young adults (*n* = 3227).

	Use Intentions			Legalization Support		
Variables	B	SE	*p*	B	SE	*p*
***Cannabis law*** (ref: no legal cannabis)						
Medical cannabis legal	−0.14	0.09	0.108	0.10	0.05	0.072
Nonmedical cannabis legal	−0.08	0.08	0.318	0.06	0.05	0.195
***Sociodemographic factors***						
Age	0.01	0.01	0.313	0.01	0.00	0.261
Male (ref: female)	0.10	0.07	0.144	0.05	0.04	0.208
Sexual minority (ref: heterosexual)	0.29	0.07	<0.001	0.23	0.04	<0.001
Hispanic (ref: non-Hispanic)	−0.05	0.09	0.527	−0.07	0.05	0.190
Race (ref: White)						
Black	−0.26	0.10	0.008	−0.07	0.06	0.224
Asian	−0.12	0.10	0.201	−0.21	0.06	<0.001
Other	−0.12	0.10	0.234	0.01	0.06	0.855
Education < Bachelor’s (ref: ≥Bachelor’s)	0.10	0.07	0.149	−0.10	0.04	0.019
Married/cohabitating (ref: single/other)	0.01	0.07	0.843	0.07	0.04	0.103
Parent/has child (ref: no)	0.05	0.08	0.539	−0.07	0.05	0.143
Community type (ref: rural)						
Micropolitan/suburban	0.02	0.09	0.840	0.11	0.05	0.032
Metropolitan/urban	0.08	0.08	0.348	0.07	0.05	0.196
***Psychosocial factors***						
PHQ-4	−0.02	0.01	0.071	0.02	0.01	0.007
ACEs	0.00	0.01	0.937	0.00	0.01	0.976
***Past-6-month cannabis message exposure***						
Promotional-message exposure	0.00	0.03	0.971	0.02	0.02	0.129
Risk-message exposure	0.06	0.03	0.060	0.01	0.02	0.516
***Perceptions of cannabis***						
Addictiveness	−0.07	0.02	<0.001	−0.04	0.01	0.002
Harm	−0.07	0.02	0.001	−0.16	0.01	<0.001
Social acceptability	0.23	0.02	<0.001	0.19	0.01	<0.001
***Past-6-month cannabis use***	3.41	0.07	<0.001	0.34	0.04	<0.001
*Adjusted R-square*		*0.613*			*0.352*	

Notes: B: Beta. SE: standard error.

**Table 5:** Multivariable linear regression models assessing sociodemographics, psychosocial factors, message exposure, and perceptions in relation to psychedelic use intentions and legalization support among US young adults (*n* = 3227).

	Use Intentions			Legalization Support		
Variables	B	SE	*p*	B	SE	*p*
***Cannabis law*** (ref: no legal cannabis)						
Medical cannabis legal	−0.06	0.05	0.262	0.00	0.06	0.943
Nonmedical cannabis legal	−0.09	0.05	0.056	0.10	0.06	0.068
***Sociodemographic factors***						
Age	0.00	0.00	0.727	0.02	0.01	0.001
Male (ref: female)	0.09	0.04	0.027	0.14	0.05	0.002
Sexual minority (ref: heterosexual)	−0.07	0.04	0.093	0.31	0.05	<0.001
Hispanic (ref: non-Hispanic)	0.03	0.05	0.594	−0.07	0.06	0.227
Race (ref: White)						
Black	0.12	0.06	0.042	−0.04	0.07	0.526
Asian	0.09	0.06	0.130	0.09	0.07	0.167
Other	−0.01	0.06	0.848	−0.03	0.07	0.659
Education < Bachelor’s (ref: ≥Bachelor’s)	0.10	0.04	0.013	0.05	0.05	0.291
Married/cohabitating (ref: single/other)	−0.01	0.04	0.799	0.02	0.05	0.649
Parent/has child (ref: no)	0.11	0.05	0.030	−0.05	0.06	0.355
Community type (ref: rural)						
Micropolitan/suburban	−0.10	0.05	0.050	0.04	0.06	0.513
Metropolitan/urban	0.01	0.05	0.884	0.11	0.06	0.057
***Psychosocial factors***						
PHQ-4	0.01	0.01	0.059	0.02	0.01	0.011
ACEs	−0.02	0.01	0.003	0.00	0.01	0.727
***Past-6-month psychedelic message exposure***						
Promotional-message exposure	0.13	0.03	<0.001	0.09	0.04	0.016
Risk-message exposure	0.13	0.03	<0.001	−0.02	0.03	0.565
***Perceptions of psychedelics***						
Addictiveness	0.00	0.01	0.880	−0.03	0.02	0.043
Harm	−0.04	0.02	0.008	−0.19	0.02	<0.001
Social acceptability	0.29	0.01	<0.001	0.15	0.01	<0.001
***Past-year psychedelic use***	1.11	0.06	<0.001	0.38	0.07	<0.001
*Adjusted R-square*		*0.413*			*0.246*	

Notes: B: Beta. SE: standard error.

## Data Availability

The datasets used and/or analyzed in the current study are available from the corresponding author upon reasonable request.

## Supplementary Materials

The following supporting information can be downloaded at: https://www.mdpi.com/article/10.3390/ijerph23020255/s1, Table S1: Pearson correlations among psychosocial factors, message exposure, perceptions, and support; Table S2: Cannabis and psychedelic perceptions, use intentions, and legalization support among US young adults, *n* = 3227.

## Abbreviations

The following abbreviations are used in this manuscript:

ACEs	Adverse childhood events
aOR	Adjusted odds ratio
B	Beta
CI	Confidence interval
CSA	Controlled Substances Act
DC	District of Columbia
DMT	Dimethyltryptamine
DOI	Diffusion of Innovation
LSD	lysergic acid diethylamide
M	Mean
MDMA	3,4-methylenedioxymethamphetamine
*p*	*p*-value
PHQ-4	Patient Health Questionnaire-4 item
PTSD	Post-traumatic stress disorder
SD	Standard deviation
SE	Standard error
THC	Tetrahydrocannabinol
US	United States
W1, W4	Wave 1, Wave 4
