# Supplementary material for: Cannabis and Psychedelics Among U.S. Young Adults: Use, Messaging Exposure, Perceptions, and Legalization Support
Source: Int J Environ Res Public Health. 2026 Feb 17;23(2):255. doi: 10.3390/ijerph23020255 (PMC12941090; doi:10.3390/ijerph23020255)
Supplement: Supplementary file 1 [file ijerph-23-00255-s001.zip › ijerph-4107768-supplementary.pdf]

**Supplementary Table S1.** Pearson correlations among psychosocial factors, message exposure, perceptions, and support

| | **Psychosocial factors** | | | **Message exposure** | | | | **Perceptions** | | | | | | **Use intentions** | | **Support** | | | |
|---|---|---|---|---|---|---|---|---|---|---|---|---|---|---|---|---|---|---|---|
| | **PHQ-4** | | | **Promotional** | | **Risk** | | **Addictiveness** | | **Harm** | | **Acceptability** | | | | **Legalization** | | **Risk minimization** | |
| | Depression | Anxiety | ACEs | Cannabis | Psychedelics | Cannabis | Psychedelics | Cannabis | Psychedelics | Cannabis | Psychedelics | Cannabis | Psychedelics | Cannabis | Psychedelics | Cannabis | Psychedelics | Cannabis | Psychedelics |
| ***Psychosocial factors*** | | | | | | | | | | | | | | | | | | | |
| PHQ-Anxiety | **.67** | 1 | | | | | | | | | | | | | | | | | |
| ACEs | **.27** | **.26** | 1 | | | | | | | | | | | | | | | | |
| ***Message exposure*** | | | | | | | | | | | | | | | | | | | |
| *Promotional* | | | | | | | | | | | | | | | | | | | |
| Cannabis | **.14** | **.15** | **.09** | 1 | | | | | | | | | | | | | | | |
| Psychedelics | **.14** | **.13** | **.08** | **.34** | 1 | | | | | | | | | | | | | | |
| *Risk* | | | | | | | | | | | | | | | | | | | |
| Cannabis | **.11** | **.09** | **.07** | **.50** | **.28** | 1 | | | | | | | | | | | | | |
| Psychedelics | **.11** | **.10** | **-08** | **.21** | **.55** | **.40** | 1 | | | | | | | | | | | | |
| ***Perceptions*** | | | | | | | | | | | | | | | | | | | |
| *Addictiveness* | | | | | | | | | | | | | | | | | | | |
| Cannabis | .01 | -.01 | **.05** | **.05** | .01 | **.06** | .01 | 1 | | | | | | | | | | | |
| Psychedelics | **-.04** | -.01 | .01 | -.01 | **-.04** | .02 | .03 | **.49** | 1 | | | | | | | | | | |
| *Harm* | | | | | | | | | | | | | | | | | | | |
| Cannabis | **-.06** | **-.07** | **-.18** | -.01 | .03 | .02 | **.05** | **.54** | **.35** | 1 | | | | | | | | | |
| Psychedelics | **-.07** | **-.07** | **-.06** | **-.05** | **-.08** | -.01 | .01 | **.38** | **.64** | **.46** | 1 | | | | | | | | |
| *Acceptability* | | | | | | | | | | | | | | | | | | | |
| Cannabis | **.10** | **.13** | **.21** | **.17** | -.01 | **-.07** | -.02 | **-.09** | **-.04** | **-.29** | **-.10** | 1 | | | | | | | |
| Psychedelics | **.12** | **.11** | **.13** | **.14** | **.17** | **.08** | **.10** | -.03 | **-.15** | **-.07** | **-.29** | **.43** | 1 | | | | | | |
| ***Use intentions*** | | | | | | | | | | | | | | | | | | | |
| Cannabis | .09 | **.10** | **.21** | **.13** | **.09** | **.13** | **.09** | **-.24** | **-.15** | **-.36** | **-.21** | **.38** | **.30** | 1 | | | | | |
| Psychedelics | .13 | **.09** | **-.08** | **.12** | **.28** | **.12** | **.23** | **-.06** | **-.15** | -.01 | **-.25** | **.13** | **.51** | **.37** | 1 | | | | |
| ***Legalization support*** | | | | | | | | | | | | | | | | | | | |
| Cannabis | **.11** | .14 | **.17** | **.12** | -.01 | **.07** | -.02 | **-.25** | **-.10** | **-.44** | **-.22** | **.46** | **.19** | **.43** | **.78** | 1 | | | |
| Psychedelics | **.11** | **.10** | **.07** | **.13** | **.13** | **.07** | **.06** | **-.16** | **-.25** | **-.18** | **-.38** | **.22** | **.34** | **.27** | **.29** | **.43** | 1 | | |

*Notes*: Bold indicates significant (p<.05).

**Supplementary Table S2.** Cannabis and psychedelic perceptions, use intentions, and legalization support among US young adults, N=3,227

| | **M (SD)** | **More** | **Equal** | **Less** |
|---|---|---|---|---|
| **Variable*** | **M (SD)** | **%** | **%** | **%** |
| ***Perceived addictiveness*** | | | | |
| Cannabis | 4.24 (1.85) | -- | -- | -- |
| Psychedelics (index score of those below) | **4.54 (1.78)** | 45.7 | 19.7 | 34.6 |
| LSD | 4.33 (2.05) | 34.4 | 32.3 | 33.3 |
| Psilocybin/amanita | 4.15 (2.03) | 30.4 | 33.7 | 45.9 |
| MDMA | **4.80 (1.94)** | 44.8 | 30.4 | 24.8 |
| Ketamine | **4.89 (1.91)** | 46.6 | 30.2 | 23.2 |
| ***Perceived harm*** | | | | |
| Cannabis | 3.55 (1.85) | -- | -- | -- |
| Psychedelics (index score of those below) | **5.12 (1.60)** | 88.1 | 15.4 | 11.9 |
| LSD | **5.13 (1.88)** | 63.4 | 25.4 | 11.2 |
| Psilocybin/amanita | **4.59 (1.98)** | 52.7 | 31.5 | 15.8 |
| MDMA | **5.40 (1.66)** | 70.2 | 22.5 | 7.3 |
| Ketamine | **5.35 (1.72)** | 67.6 | 23.8 | 8.6 |
| ***Perceived social acceptability*** | | | | |
| Cannabis | 5.22 (1.93) | -- | -- | -- |
| Psychedelics (index score of those below) | **2.73 (1.66)** | 4.3 | 13.2 | 82.4 |
| LSD | **2.72 (1.82)** | 3.3 | 18.0 | 78.7 |
| Psilocybin/amanita | **3.13 (1.95)** | 4.2 | 21.6 | 74.1 |
| MDMA | **2.67 (1.83)** | 3.7 | 17.4 | 78.9 |
| Ketamine | **2.42 (1.74)** | 4.0 | 16.5 | 79.5 |
| ***Next-year use intentions*** | | | | |
| Cannabis | 3.34 (2.51) | -- | -- | -- |
| Psychedelics (index score of those below) | **1.55 (1.21)** | 5.5 | 43.9 | 50.1 |
| LSD | **1.51 (1.30)** | 3.0 | 47.5 | 49.4 |
| Psilocybin/amanita | **1.76 (1.57)** | 5.0 | 48.2 | 46.8 |
| MDMA | **1.48 (1.28)** | 2.9 | 47.9 | 48.2 |
| Ketamine | **1.45 (1.26)** | 3.6 | 46.4 | 50.0 |
| ***Legalization support*** | | | | |
| Cannabis | 3.88 (1.18) | -- | -- | -- |
| Psychedelics | **2.83 (1.24)** | 8.0 | 26.0 | 66.0 |

*Notes:* *1=not at all to 7=extremely. Bold M and SD indicates significantly different vs. cannabis. Each psychedelic perception and use intention were significantly correlated (p's<.05). % were yielded by subtracting psychedelic from cannabis measures and identifying % reporting less, equal, or more/higher.
